# Reynoutrin Improves Ischemic Heart Failure in Rats Via Targeting S100A1

**DOI:** 10.3389/fphar.2021.703962

**Published:** 2021-07-23

**Authors:** Wenkai Yang, Hanjian Tu, Kai Tang, Haozhong Huang, Shi Ou, Jianguo Wu

**Affiliations:** ^1^ Department of Cardiovascular Surgery, Central People’s Hospital of Zhanjiang, Zhanjiang, China; ^2^ Department of Cardiac Surgery, Shanghai East Hospital, Tongji University School of Medicine, Shanghai, China

**Keywords:** reynoutrin, ischemic heart failure, myocardial fibrosis, inflammation, S100A1

## Abstract

This study investigated the effects of reynoutrin on the improvement of ischemic heart failure (IHF) and its possible mechanism in rats. The rat heart failure model was established by permanently ligating the left anterior descending coronary artery (LAD) and administering different doses of reynoutrin. Cardiac function, inflammatory factors releasing, oxidative stress, cardiomyocytes apoptosis, and myocardial fibrosis were evaluated. Western blotting was used to determine protein expression levels of S100 calcium-binding protein A1 (S100A1), matrix metallopeptidase 2(MMP2), MMP9, phosphorylated (p-) p65, and transforming growth factor -β1 (TGF-β1) in myocardial tissue of the left ventricle. Results showed that reynoutrin significantly improved cardiac function, suppressed the release of inflammatory factors, reduced oxidative stress, inhibited cardiomyocytes apoptosis, and attenuated myocardial fibrosis in rats with IHF. In rat myocardial tissue, permanent LAD-ligation resulted in a significant down-regulation in S100A1 expression, whereas reynoutrin significantly up-regulated S100A1 protein expression while down-regulating MMP2, MMP9, p-p65, and TGF-β1 expressions. However, when S100A1 was knocked down in myocardial tissue, the above-mentioned positive effects of reynoutrin were significantly reversed. Reynoutrin is a potential natural drug for the treatment of IHF, and its mechanism of action involves the up-regulation of S100A1 expression, thereby inhibiting expressions of MMPs and the transcriptional activity of nuclear factor kappa-B.

## Introduction

Heart failure is a myocardial injury caused by several factors, such as myocardial infarction, hypertension, and myocarditis. Its main clinical feature is the attenuation of the heart’s pumping capacity, resulting in insufficient blood to meet the body’s metabolic needs. Ischemic heart failure (IHF) is a type of heart failure caused by short-term or continuous interruption of coronary circulatory perfusion and insufficient blood supply to the myocardium due to blood flow obstruction. Coronary artery disease due to atherosclerosis is a common cause of IHF, which is often accompanied by an elevation in the ST-segment of an electrocardiogram (ECG) (Sekulic et al., 2019). Myocardial injury, regardless of the causes, always results in myocardial inflammation. The body’s anti-inflammatory response triggers pro-fibrotic signals, leading to myocardial fibrosis (Frangogiannis, 2008; Yajima and Knowlton, 2009). The hallmark of pathological fibrosis is the excessive deposition of extracellular matrix (ECM) proteins and key pro-fibrotic stimuli, such as matrix metallopeptidase proteins (MMPs) and transforming growth factors (TGFs), which greatly contribute to the progression of myocardial fibrosis (Kong et al., 2014). The global morbidity and mortality rates of IHF are rising, posing a serious public health threat. Despite many breakthroughs in heart failure research, effective treatment strategies for IHF in clinical practice are still inadequate. Therefore, the development of new drugs and methods for the treatment of IHF is imperative.

**FIGURE 1:**
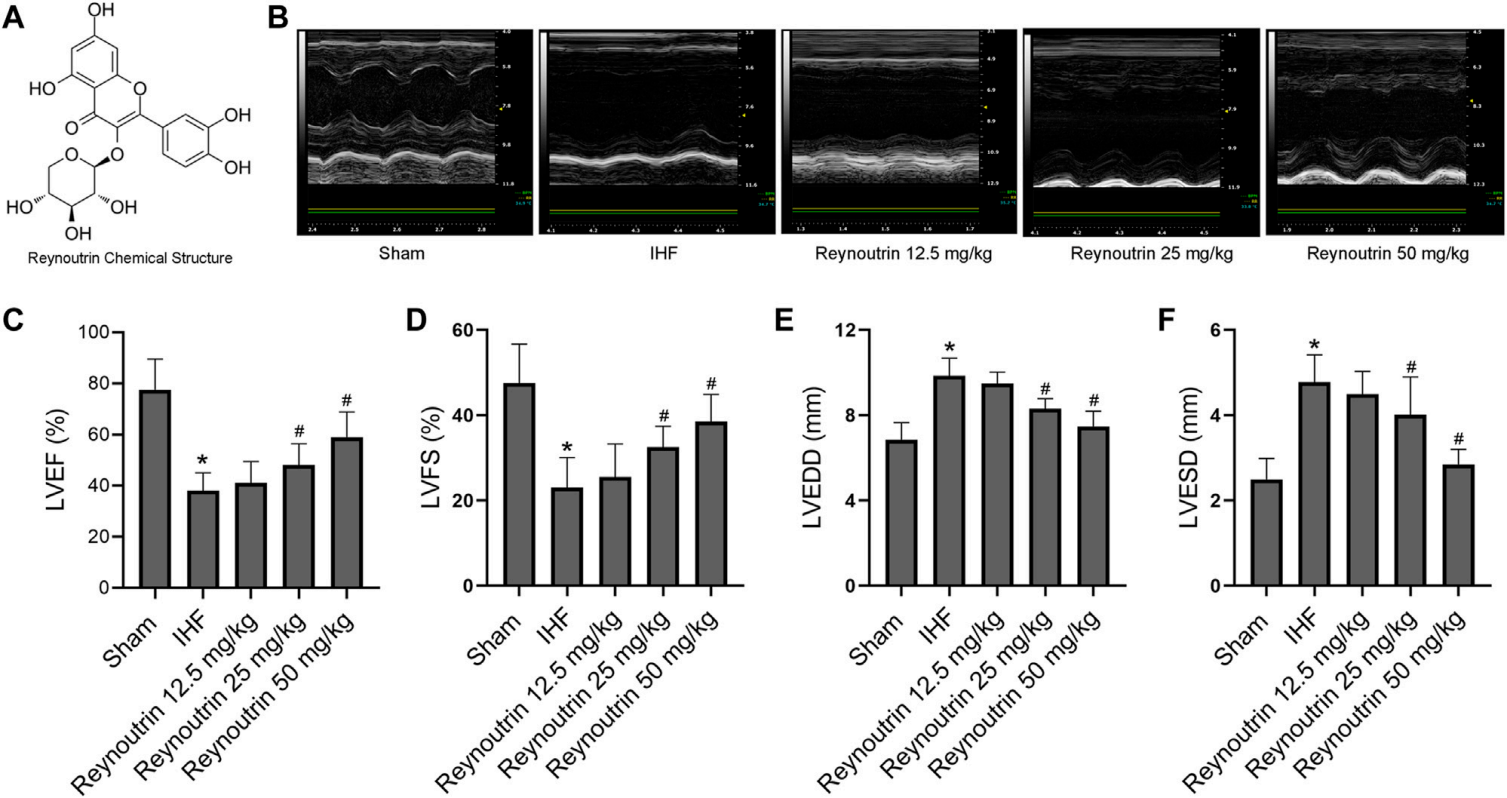
Effects of reynoutrin on cardiac function of rats. **(A)** The chemical structure of reynoutrin (PubChem CID: 5320861). **(B)** The representative image of echocardiogram in each group. **(C)** LVEF histogram of each experimental group. **(D)** LVFS histogram of each experimental group. **(E)** LVEDD histogram of each experimental group. **(F)** LVESD histogram of each experimental group. The values were presented as the means ± standard error in each experiment group (n = 10). ^*^ *p* < 0.05 vs. Sham group; ^#^ *p* < 0.05 vs. IHF group.

Reynoutrin (Figure 1) is a natural flavonoid that exists in the leaves and fruits of a variety of natural plants and has been shown to have potential antioxidant and antiviral properties (Ho et al., 2012; Rehman et al., 2018; Butkevičiūtė et al., 2020; Santos et al., 2021). In the preliminary experiments, we found that reynoutrin can effectively improve cardiac function in rats with IHF caused by permanent left anterior descending coronary artery (LAD) ligation, as well as significantly up-regulate the protein expression of S100 calcium-binding protein A1 (S100A1) in myocardial tissue. S100A1 is a Ca^2+^ binding protein that belongs to the S100 protein family (Most et al., 2001). Studies have shown that S100A1 protein levels in the heart tissue of patients with end-stage heart failure are reduced, and the lack of S100A1 accelerates heart failure in laboratory animals (Soltani et al., 2021). S100A1 plays an important role in the occurrence and development of heart failure. This study investigated the anti-IHF effect of reynoutrin as well as the relationship between its mechanism of action and S100A1.

## Materials and Methods

### Reagents

Reynoutrin was purchased from Macklin (Shanghai, China) and dissolved in dimethylsulfoxide (DMSO) (20 mg/ml). Antibodies of S100A1, matrix metallopeptidase 2 (MMP2), MMP9, transforming growth factor -β1 (TGF-β1) and GAPDH were purchased from Proteintech (Rosemont, IL, United States). Antibodies of p65 and phosphorylated (p-) p65 were purchased from Abcam (Cambridge, United Kingdom). ELISA detection kits for tumor necrosis factor *α* (TNF-α) and interleukin 6 (IL-6) were purchased from Proteintech (Rosemont, IL, United States). Malondialdehyde (MDA) and total superoxide dismutase (SOD) detection kits were purchased from Nanjing Jiancheng Bioengineering Institute (Nanjing, China).

### Measurement of Cytotoxicity

Rat cardiomyocytes H9c2 purchased from Procell (Wuhan, CHN), and cultured in DMEM with 10% fetal bovine serum. The cytotoxicity of reynoutrin on H9c2 was evaluated by 3-(4,5-dimethylthiazol-2-yl)-5-(3-carboxymethoxyphenyl)-2-(4-sulfophenyl)-2H-tetrazolium (MTT; Promega, WI, United States) assay. Briefly, after 24 h treatment with different concentrations of reynoutrin in 96-well plates, the H9c2 cells were incubated with 20 μl MTT (5 mg/ml) in 100 μlcell culture medium for 4 h at 37°C, and the absorbance of each well was measured at a wavelength of 490 nm.

### Animals

Specific-pathogen-free male *Sprague Dawley* rats (180–220 g) were obtained from the Experimental Animal Center of Guangdong Medical University, and they were usually kept in a 22–25 C constant temperature room with an electric lamp to simulate natural light. Experiments were carried out according to the Guide for the Care and Use of Laboratory Animals published by the U.S. National Institutes of Health (NIH Publication No.85–23, revised in 1996), and have been approved by the Ethics Committee.

### Construction of Recombinant Lentivirus

The RNA interference target sequences were designed based on S100A, and the single-stranded DNA oligo containing interference sequences was synthesized. An annealing reaction was performed to obtain double-stranded DNA, which was then ligated to the digested lentiviral vector. The product was transformed into *Escherichia coli* competent cells and the positive recombinant was determined using PCR. Next, the insert sequence was verified and the plasmids were purified. Homologous recombination was performed to generate recombinant lentivirus and 293 T cells were used to amplify the virus particles. The virus particles were harvested from the supernatant by density gradient centrifugation and then stored at −80 C. The interference efficiency of S100A1 was confirmed by fluorescence observation from green fluorescent protein (GFP) and western blotting (Supplementary Figure 2).

### Experimental Grouping and Treatment

The rats were randomly divided into the following groups: 1) Normal control group (NC), in which the rats were injected intraperitoneally with 0.2 ml/rat/day vehicle for 35 days. 2) Reynoutrin low-, medium-, and high-dose intervention groups, in which the rats were injected intraperitoneally with 12.5, 25, and 50 mg/kg reynoutrin respectively, for 35 days. 3) Sham control group (Sham), in which the rats were injected intraperitoneally with 0.2 ml/rat/day vehicle for 7 days, followed by exposure of the LAD by thoracotomy 1 hour after the seventh injection. Rats received subcutaneous injections of 0.1 mg/kg buprenorphine for analgesia after surgery, once every 12 h, for three consecutive days (Foley et al., 2019). In addition, 1 hour after the operation, the rats were injected with 0.2 ml/rat/day vehicle for 28 consecutive days. 4) Ischemic heart failure group (IHF), in which the rats were injected intraperitoneally with 0.2 ml/rat/day vehicle for 7 days, and permanent LAD-ligation was performed 1 hour after the seventh injection. As previously described (Bacmeister et al., 2019), the rats were anesthetized with pentobarbital sodium (50 mg/kg) intraperitoneally and mechanically ventilated. The heart was dissected and exposed in the third left intercostal space, and sutures were used to permanently ligate the left anterior descending coronary artery. During this time, whitening of the distal myocardial tissue can be observed, and there is an elevation in the ST segment of the ECG. Rats received buprenorphine subcutaneous injections for analgesia after surgery as above described. One hour after the operation, the rats were injected with 0.2 ml/rat/day vehicle for 28 consecutive days. 5) Reynoutrin low-, medium-, and high-dose treatment groups, in which the rats were injected intraperitoneally with 12.5, 25, and 50 mg/kg reynoutrin, respectively, for 7 days, with the permanent LAD-ligation performed as described above 1 h after the seventh injection. One hour after the operation, the corresponding doses of reynoutrin were injected intraperitoneally for 28 consecutive days. 6) Reynoutrin combined with S100A1 knock-down group (Reynoutrin + shS100A1), in which 2 × 10^7^ transduction units of lentiviral vectors encoding S100A1 interfering RNA were injected into the left ventricular wall of rats 1 week before LAD-ligation. Based on a previously described method (Liu et al., 2018), the rats were anesthetized by intraperitoneal injection of pentobarbital sodium (50 mg/kg) and mechanically ventilated. In order to expose the heart, dissection was performed in the third left intercostal space, and then lentiviral vectors were injected at multiple points in the myocardium of the left ventricle using a micro-syringe (Hamilton, Reno, NV, United States). The rats were pretreated with reynoutrin daily and LAD-ligation was performed 1 hour after the seventh reynoutrin injection, with reynoutrin treatment continuing for 28 consecutive days postoperatively. In addition, 1 week after lentivirus infection, the left ventricular myocardial tissue was taken for frozen section and extensive expression of green fluorescent protein (GFP) was observed under a fluorescence microscope, indicating that the virus infection was successful, as shown in Supplementary Figure 1. Subsequently, observations were carried out once a week, and stable expression of FGP was observed. 7) S100A1 knock-down group (shS100A1), in which the rats were treated similarly to the Reynoutrin + shS100A1 group, but vehicle instead of reynoutrin. Taking into account the high mortality rate caused by LAD-ligation, each experimental group was allocated 20–25 rats to ensure that at least 10 rats in each experimental group survived.

### Assessment of Cardiac Function

The rats were anesthetized with pentobarbital sodium 12 h after the last administration, and the myocardial contractility of the rats in each group was evaluated using the Vevo2100 imaging system (VisualSonics, Toronto, ON, Canada), and the left ventricular end-diastolic diameter (LVEDD), the left ventricular end-systolic diameter (LVESD), the left ventricular fractional shortening (LVFS) and the left ventricular ejection fraction (LVEF) were measured.

### Measurement of Myocardial Infarct Size

Tissue death was determined using triphenyltetrazolium chloride (TTC, Sigma-Aldrich, United States) staining. The hearts of the rats were completely removed and quickly frozen with liquid nitrogen. Following that, the heart was sectioned at the point of 2 mm below the ligation point with a thickness of less than 1 mm and soaked in 1% TTC at 37°C for 10–15 min. After fixation with 10% formaldehyde, the infarct area was white, while the normal area was red. After images were taken, the semi-quantitatively infarct size of the particular section of left ventricular was calculated using the Image Pro Plus 6.0 software (Media Cybernetics, Bethesda, MD, United States).

### Assessment of Inflammatory Factors and Oxidative Stress

The obtained peripheral blood was centrifuged to obtain serum, and the levels of TNF-α and IL-6 in serum were detected by ELISA detection kits. The left ventricular myocardial tissue was thoroughly ground with liquid nitrogen and dissolved with normal saline, and the levels of SOD and MDA in the homogenate were detected by detection kits. The above-mentioned testing operations were strictly performed in accordance with the instructions provided by the manufacturers.

### Tunel Staining

Terminal deoxynucleotidyl transferase-mediated deoxyuridine triphosphate-biotin nick end labeling (Tunel) was used to evaluate the apoptosis of cardiomyocytes. Briefly, the myocardial paraffin sections were incubated with 10 mM proteinase K for 15 min, then incubated with the TUNEL reaction mixture at a constant temperature of 37 C in the dark for 60 min, and finally incubated with DAPI for 10 min. The staining results were observed under a fluorescence microscope, and the apoptotic cardiomyocyte nucleus was green. Five regions were randomly selected in each section to count the apoptotic cells.

### Masson Staining

The paraffin sections were obtained from the myocardial tissues of rats in each group after formaldehyde fixation and paraffin embedding, and then the Masson staining was performed. Collagen deposition was observed under a microscope and photographed. Image Pro Pius 6.0 software (Media Cybernetics, Rockville, MA, United States) was used to quantitatively analysis of Masson staining images.

### Western Blotting

Proteins were extracted from left ventricular myocardial tissue by using radioimmunoprecipitation assay (RIPA) lysis buffer and size fractionated by SDS polyacrylamide gel electrophoresis. Membranes were incubated with target antibodies at 4 C overnight. Then membranes were incubated with the horseradish peroxidase-conjugated secondary antibodies for 2 h at room temperature after washed by tris-buffered saline and tween 20. The immune complexes were visualized by enhanced chemiluminescence after washing again and the band intensity was measured quantitatively and analyzed with the Image J v2.1.4.7 software (National Institutes of Health, Bethesda, MD, United States).

### Immunofluorescence Staining

As previously reported, paraffin sections of the left ventricular myocardial tissue were incubated with bovine serum albumin, followed by incubation with anti-TGF-β1 (1:400) overnight at 4°C. The sections were then washed and incubated with Texas red conjugated anti-mouse IgG (1:200; Abcam) for 1 h in the dark. After further washing, the sections were stained with DAPI for 5 min, washed, and mounted. An inverted fluorescence microscope (Leica, Wetzlar, GER) was used to observe the samples and capture images, and images were analyzed using the Image Pro Plus 6.0 software (Media Cybernetics, Bethesda, MD, United States).

### Statistical Analysis

All data were presented as the mean ± standard error of the mean and analyzed using SPSS version 20.0 (IBM Corp., Armonk, NY, United States). Shapiro—Wilk was used to test the assumption of normality for normality prior to additional analysis. Student’s unpaired, two—tailed t—test (2 groups), or one - or two—way ANOVA (>2 groups) were used to analyze the normally distributed data followed by Dunnett’s or Tukey’s *post*—*hoc* test. A *p*-value < 0.05 was considered to be statistically significant.

## Results

### The Cytotoxicity Assessment of Reynoutrin on H9c2 Cells

**FIGURE 2:**
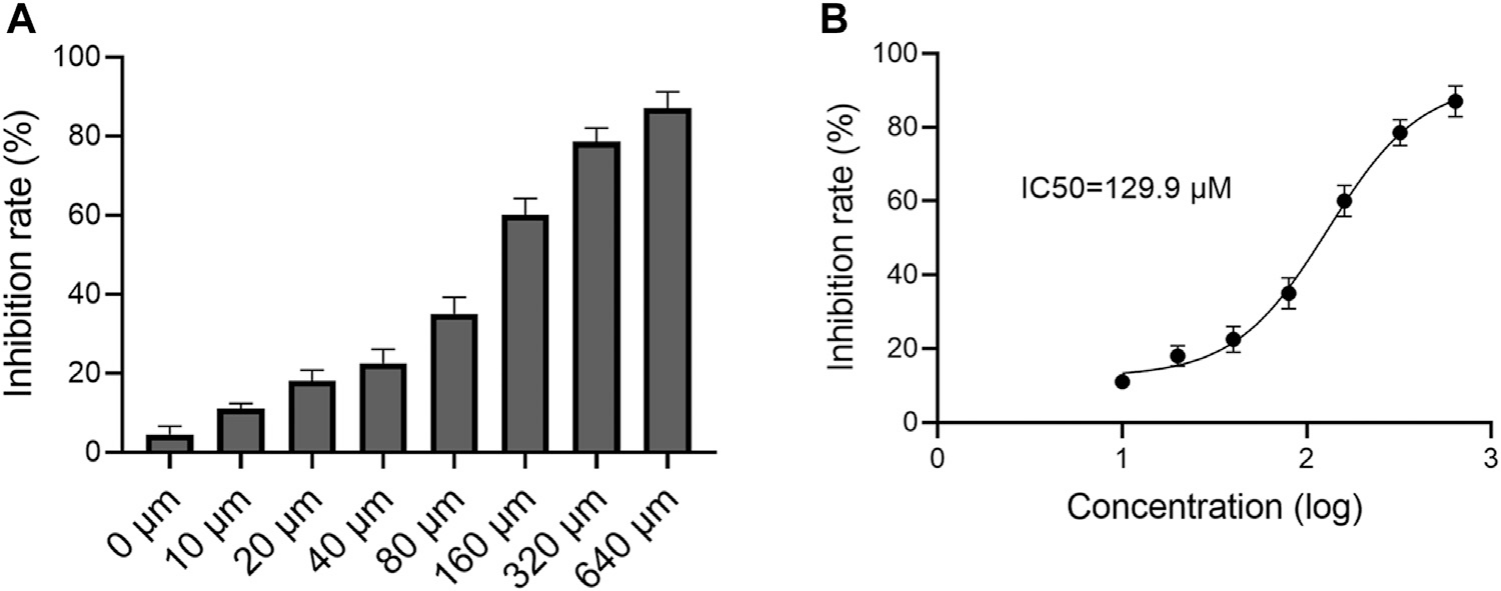
Effect of reynoutrin on H9c2 cells viability. MTT was used to detect the cytotoxicity of reynoutrin on H9c2 cells. **(A)** The inhibition rate of different concentrations of reynoutrin on H9c2 cells for 24 h **(B)** Logarithmic function dose-effect curve of reynoutrin on H9c2 cells. The values were presented as the means ± standard error for six independent experiments

As shown in Figure 2, the inhibitory effect of reynoutrin on H9c2 cells showed an obvious dose and time dependence. The calculated IC50 value of reynoutrin in H9c2 cells was 129.9 μM > 100 μM (Garifullin et al., 2019), suggesting that the cytotoxicity of reynoutrin could be ignored.

### The Effects of Reynoutrin on Cardiac Function in Rats With Ischemic Heart Failure

As shown in Table 1, different doses of reynoutrin had no significant effect on LVEF, LVFS, LVEDD, and LVESD in normal rats (*p* > 0.05 vs. NC group). Permanent LAD-ligation resulted in significant decreases in LVEF and LVFS, while significantly increasing LVEDD and LVESD in rats (*p* < 0.05 vs. Sham group), as seen in Figures 1B–F. Different doses of reynoutrin improved abnormal cardiac function in rats caused by LAD-ligation to varying degrees. At reynoutrin doses of 25 and 50 mg/kg, the LVEF and LVFS of the rats were significantly increased, while their LVEDD and LVESD were significantly decreased when compared to the IHF rats (*p* < 0.05). It was worth noting that 50 mg/kg reynoutrin displayed the most obvious improvement.

**TABLE 1:** The effects of reynoutrin on normal rats (means ± S.E.M).

Groups	LVEF (%)	LVFS (%)	LVEDD (mm)	LVESD (mm)	TNF-α (pg/ml)	IL-6 (pg/ml)	SOD (U/mg protein)	MDA (mmol/mg protein)
NS (n = 6)	77.6 ± 5.2	47.3 ± 2.1	6.7 ± 0.3	2.5 ± 0.1	21.2 ± 3.7	23.4 ± 4.8	21.2 ± 3.7	3.7 ± 0.2
Reynoutrin 12.5 mg/kg (n = 6)	78.1 ± 5.7	47.9 ± 1.8	6.7 ± 0.5	2.6 ± 0.0	20.4 ± 4.8	24.1 ± 3.7	20.4 ± 4.8	3.7 ± 0.2
Reynoutrin 25 mg/kg (n = 6)	81.9 ± 6.7	48.3 ± 2.2	6.6 ± 0.3	2.5 ± 0.1	19.6 ± 2.4	22.6 ± 2.3	19.6 ± 2.4	3.6 ± 0.1
Reynoutrin 50 mg/kg (n = 6)	82.4 ± 7.1	49.2 ± 2.5	6.6 ± 0.4	2.5 ± 0.1	19.1 ± 2.7	21.4 ± 1.9	19.1 ± 2.7	3.5 ± 0.2

### The Effects of Reynoutrin on the Levels of Inflammatory Factors and Oxidative Stress in Rats With Ischemic Heart Failure

**FIGURE 3:**
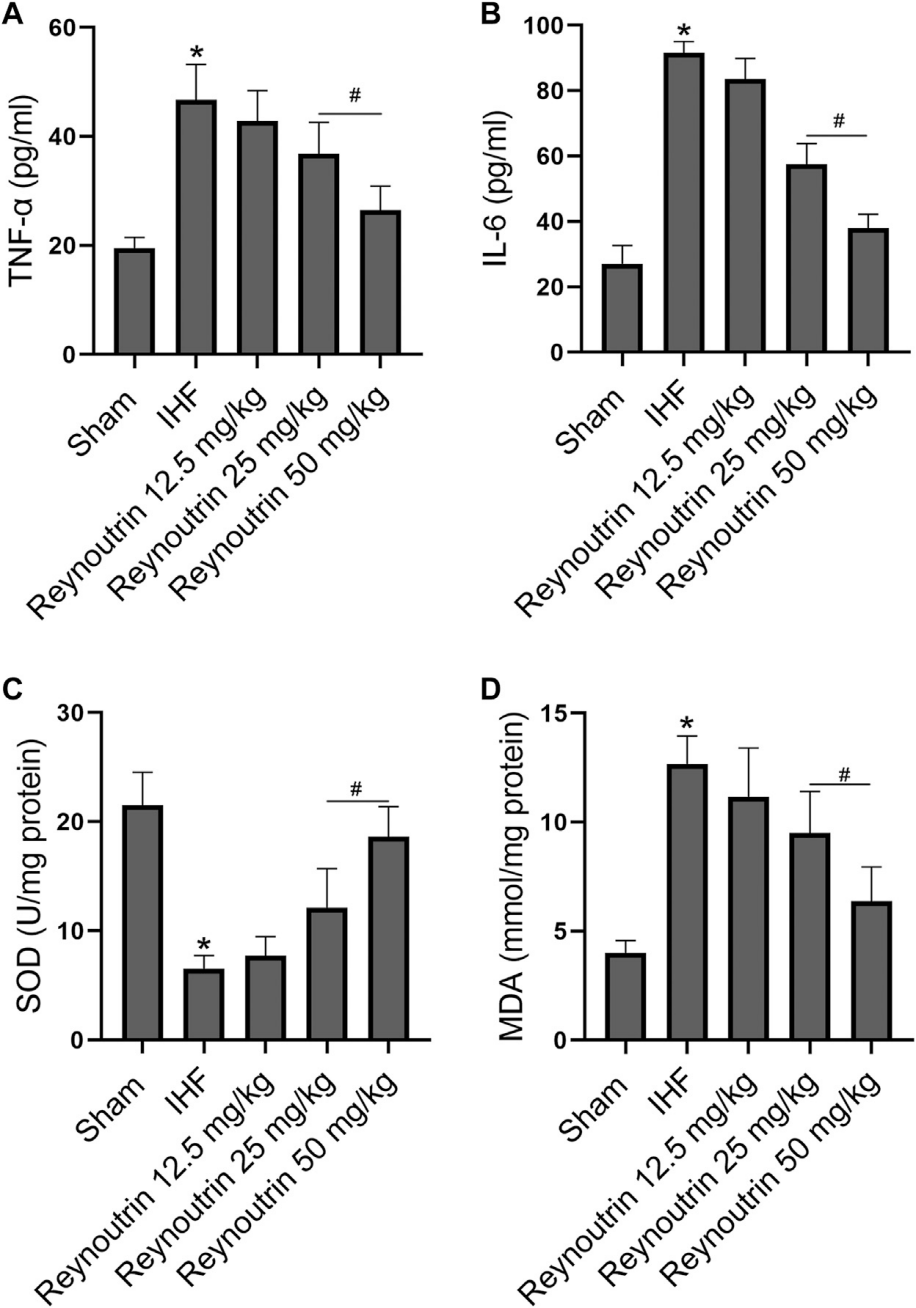
Effects of reynoutrin on the levels of TNF-α, IL 6, SOD and MDA in rats. **(A)** The concentration of TNF-α in serum of each group. **(B)** The concentration of IL six in serum of each group. **(C)** The concentration of SOD in myocardium of each group. **(D)** The concentration of MDA in myocardium of each group. The values presented as the means ± standard error in each experiment group (*n* = 10). ^*^ *p* < 0.05 vs. Sham group; ^#^ *p* < 0.05 vs. IHF group.

Different doses of reynoutrin had no significant effect on serum inflammatory factor levels (TNF-α and IL-6) and myocardial oxidative stress levels (SOD and MDA) in normal rats (*p* > 0.05 vs. NC group), as seen in Table 1. As shown in Figures 3A,B, permanent LAD-ligation led to a significant increase in serum TNF-α and IL-6 in rats (*p* < 0.05 vs. Sham group). Different doses of reynoutrin treatments were able to suppress the levels of inflammatory factors in rats with IHF in a dose-dependent manner. Reynoutrin had the most significant effect on inhibiting the release of inflammatory factors at 50 mg/kg. In the left ventricular myocardium of the rats, LAD-ligation resulted in a significant decrease in SOD level and a significant increase in MDA level (*p* < 0.05 vs. Sham group). Furthermore, different doses of reynoutrin could inhibit the oxidative stress in the myocardium tissue of rats with IHF in a dose-dependent manner. The effect of reducing oxidative stress was most obvious when reynoutrin was administered at 50 mg/kg. In subsequent experiments, reynoutrin was consistently used at 50 mg/kg.

### S100A1 Knock-Down Attenuated the Effect of Reynoutrin on Improving Cardiac Function in Rats With Ischemic Heart Failure

**FIGURE 4:**
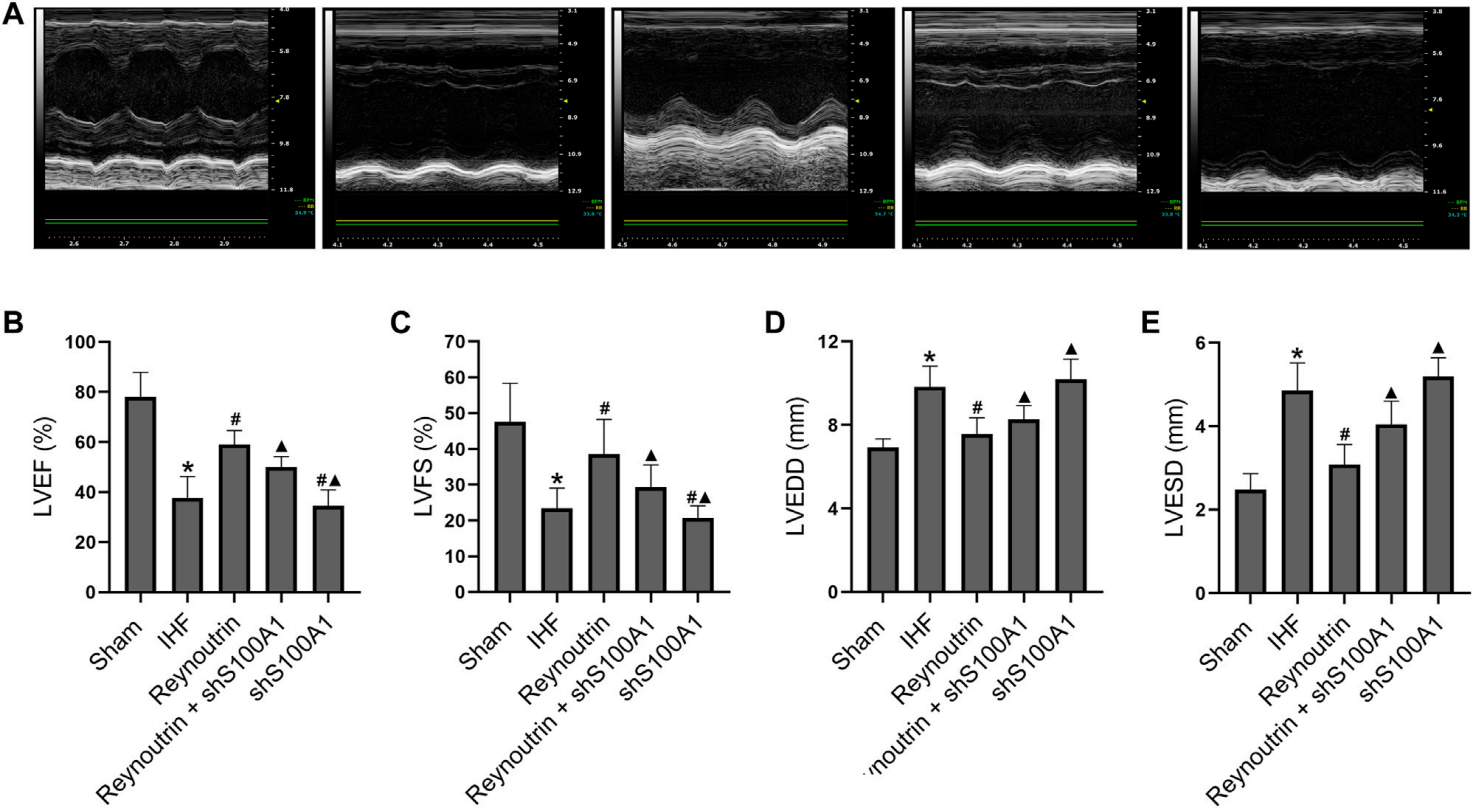
Effects of reynoutrin and S100A1 knock-down on cardiac function of rats. The dose of reynoutrin here was 50 mg/kg. **(A)** The representative images of echocardiogram of each group. **(B)** LVEF histogram of each experimental group. **(C)** LVFS histogram of each experimental group. **(D)** LVEDD histogram of each experimental group. **(E)** LVESD histogram of each experimental group. The values presented as the means ± standard error in each experiment group (*n* = 10). ^*^ *p* < 0.05 vs. Sham group; ^#^ *p* < 0.05 vs. IHF group; ^▲^ *p* < 0.05 vs. Reynoutrin group.

As shown in Figure 4, permanent LAD-ligation resulted in significant decreases in LVEF and LVFS, as well as significant increases in LVEDD and LVESD in rats (*p* < 0.05 vs. Sham group). On the other hand, reynoutrin treatment significantly increased LVEF and LVFS, while decreasing LVEDD and LVESD in rats with IHF (*p* < 0.05 vs. IHF group). However, when S100A1 was knocked down in the myocardial tissue of rats, the effect of reynoutrin on improving the cardiac function in IHF rats was significantly reversed (*p* < 0.05 vs. Reynoutrin group). In addition, S100A1 knock-down alone could significantly aggravate heart failure in rats with permanent LAD-ligation (*p* < 0.05 vs. IHF group).

### S100A1 Knock-Down Attenuated the Effects of Reynoutrin on Improving Inflammation Response and Oxidative Stress in Rats With Ischemic Heart Failure

**FIGURE 5:**
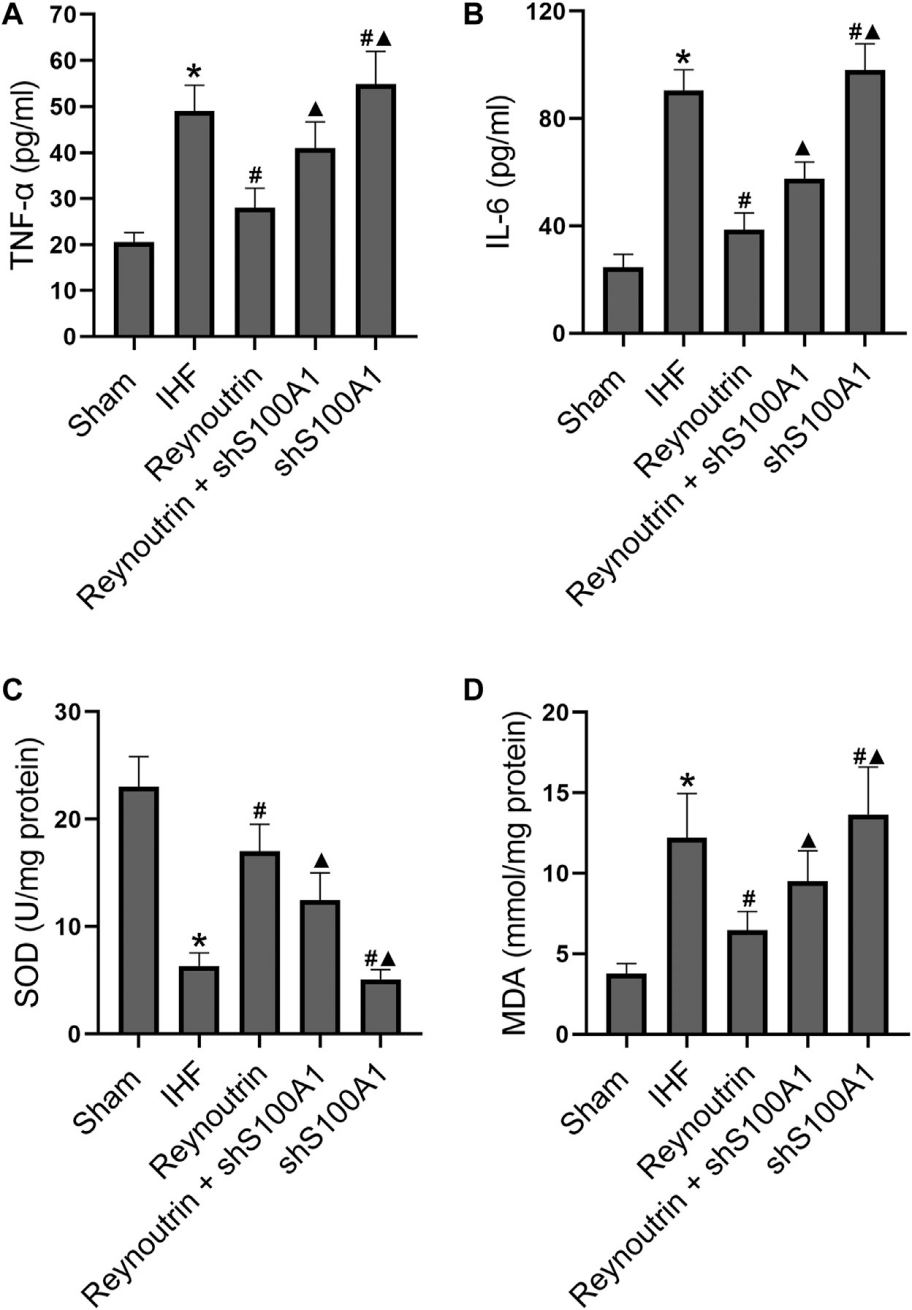
Effects of reynoutrin and S100A1 knock-down on the levels of TNF-α, IL 6, SOD and MDA in rats. The dose of reynoutrin here was 50 mg/kg. **(A)** The concentration of TNF-α in serum of each group. **(B)** The concentration of IL six in serum of each group. **(C)** The concentration of SOD in myocardium of each group. **(D)** The concentration of MDA in myocardium of each group. The values presented as the means ± standard error in each experiment group (*n* = 10). ^*^ *p* < 0.05 vs. Sham group; ^#^ *p* < 0.05 vs. IHF group; ^▲^ *p* < 0.05 vs. Reynoutrin group.

Permanent LAD-ligation caused a significant increase in serum TNF-α and IL-6 in rats (*p* < 0.05 vs. Sham group), while reynoutrin treatment significantly inhibited the releases of inflammatory factors (*p* < 0.05 vs. IHF group), as seen in Figure 5 A and B. However, when S100A1 in rat myocardial tissue was knocked down, the effect of reynoutrin on inhibiting the release of inflammatory factors in IHF rats was significantly reversed (*p* < 0.05 vs. Reynoutrin group). In addition, the knock-down of S100A1 alone could significantly increase the levels of inflammatory factors in rats with IHF (*p* < 0.05 vs. IHF group).

In the myocardial tissue of rats with IHF, the level of SOD was significantly decreased, while the level of MDA was significantly increased (*p* < 0.05 vs. Sham group), as shown in Figures 5 C,D. Furthermore, the administration of reynoutrin significantly increased SOD and inhibited MDA (*p* < 0.05 vs. IHF group). The abovementioned effects of reynoutrin were significantly reversed (*p* < 0.05 vs. Reynoutrin group) when S100A1 in cardiomyocytes was knocked down. In addition, the knock-down of S100A1 alone was found to significantly elevate the oxidative stress level in the myocardium of rats with LAD-ligation (*p* < 0.05 vs. IHF group).

### Reynoutrin Improved Infarction, Fibrosis, and Apoptosis in the Myocardium of Rats With Ischemic Heart Failure

**FIGURE 6:**
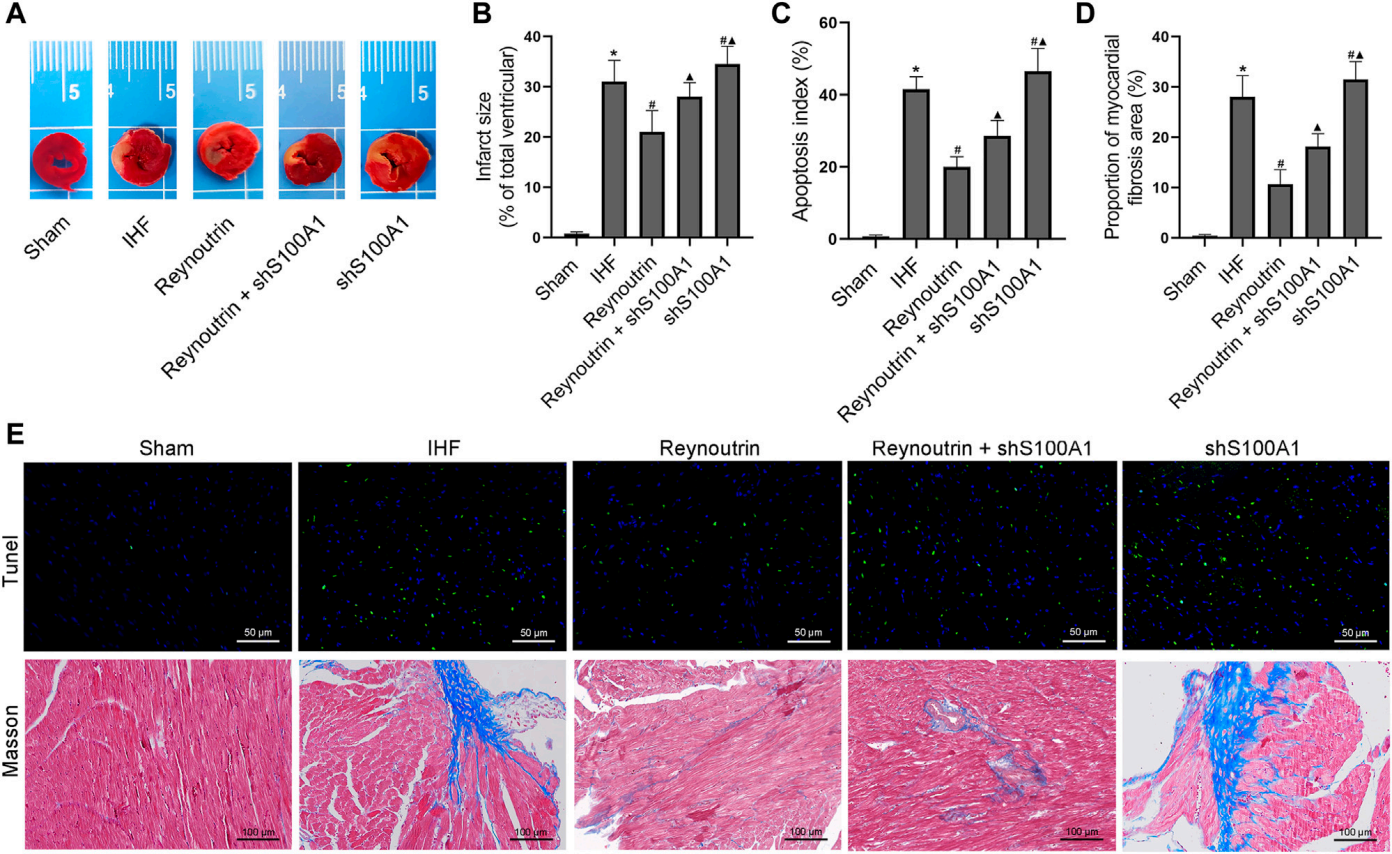
Effects of reynoutrin and S100A1 knock-down on infarction, fibrosis and apoptosis of myocardium in rats. The dose of reynoutrin here was 50 mg/kg. **(A)** The representative images of TTC staining of each group. **(B)** The histogram of infarct size in each experimental group, which in percent of the surface of the stained slices. **(C)** The histogram of apoptosis index in each experimental group. **(D)** The histogram of myocardial fibrosis area proportion in each experimental group. **(E)** The representative images of Masson and Tunel staining of each group. The values presented as the means ± standard error in each experiment group (*n* = 10). ^*^ *p* < 0.05 vs. Sham group; ^#^ *p* < 0.05 vs. IHF group; ^▲^ *p* < 0.05 vs. Reynoutrin group.

The area of myocardial infarction was evaluated using TTC staining while the level of myocardial apoptosis was determined using Tunel staining. Masson staining was used to evaluate collagen deposition in myocardial tissue. As illustrated in Figure 6, permanent LAD-ligation resulted in a significant myocardial infarction, myocardial apoptosis, and myocardial fibrosis in rats as compared to the sham group. Reynoutrin treatment significantly reduced the area of myocardial infarction, inhibited cardiomyocyte apoptosis, and improved myocardial fibrosis in rats with IHF. However, the abovementioned positive effects of reynoutrin were significantly attenuated after S100A1 was knocked down in myocardial tissue. Furthermore, the knock-down of S100A1 alone significantly increased the area of myocardial infarction, the level of cardiomyocytes apoptosis, and the degree of myocardial fibrosis in IHF rats.

### The Effects of Reynoutrin and S100A1 Knock-Down on Protein Expressions in Myocardial Tissue of Rats With Ischemic Heart Failure

**FIGURE 7:**
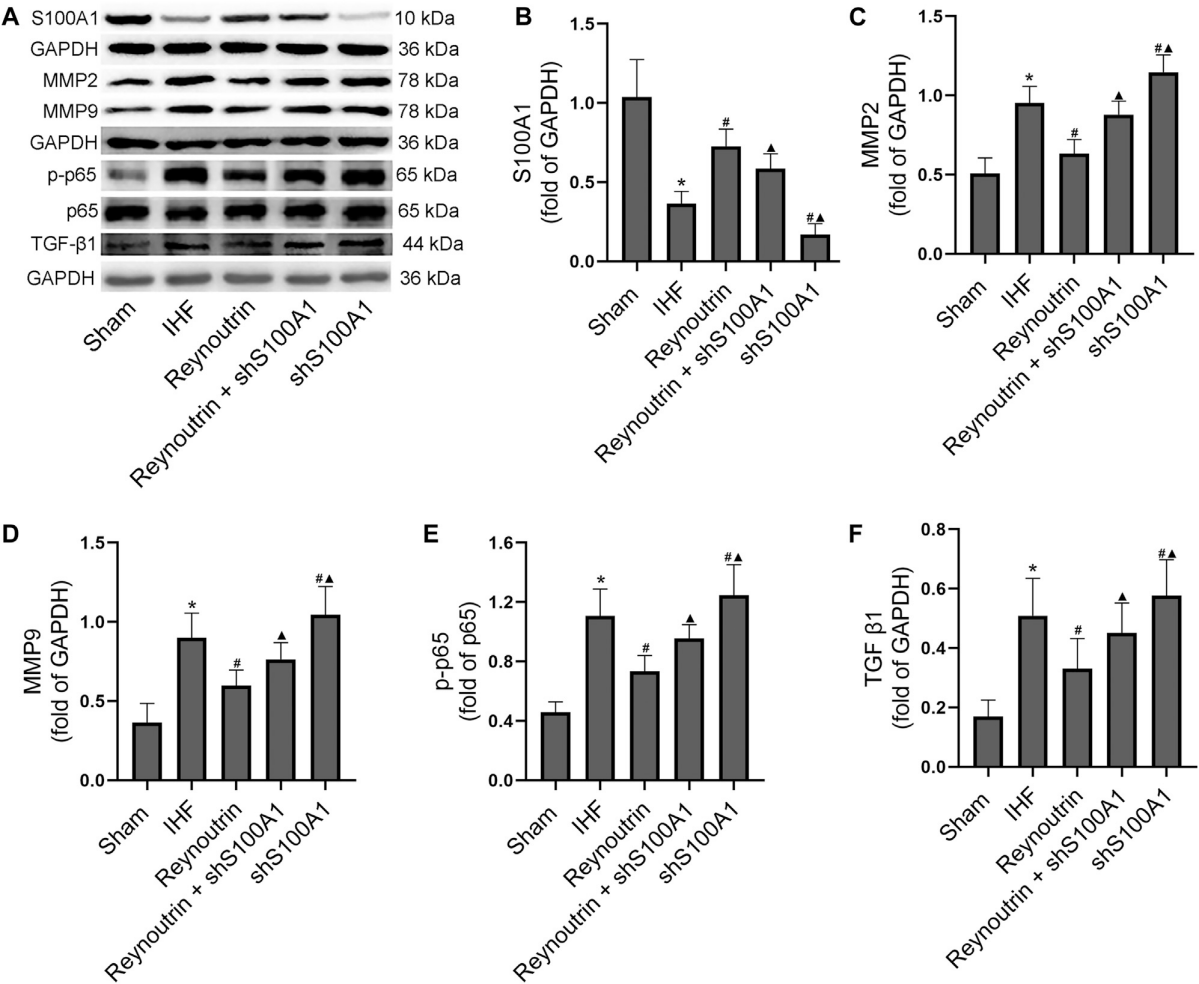
Effects of reynoutrin and S100A1 knock-down on protein expressions in myocardial tissue of rats. The dose of reynoutrin here was 50 mg/kg. **(A)** The representative blots of S100A1, MMP2, MMP9, p-p65, p65, TGF-β1 and GAPDH in each group. **(B)** Semi quantitative analysis of S100A1 in each group. **(C)** Semi quantitative analysis of MMP2 in each group. **(D)** Semi quantitative analysis of MMP9 in each group. **(E)** Semi quantitative analysis of p-p65 in each group. **(F)** Semi quantitative analysis of TGF-β1 in each group. The values presented as the means ± standard error in each experiment group (*n* = 10). ^*^ *p* < 0.05 vs. Sham group; ^#^ *p* < 0.05 vs. IHF group; ^▲^ *p* < 0.05 vs. Reynoutrin group.

The expression levels of S100A1, MMP2, MMP9, p-p65, and TGF-β1 in left ventricular myocardial tissue of rats were detected using Western blotting, as shown in Figure 7. Compared to the sham operation group, the expression of S100A1 protein in the myocardial tissue of rats in the IHF group was significantly down-regulated (*p* < 0.05), whereas reynoutrin intervention significantly up-regulated the level of S100A1 protein in the myocardial tissue of rats with IHF (*p* < 0.05 vs. IHF group). S100A1 knock-down inhibited the expression of S100A1 in rat myocardial tissue and significantly reversed the effect of reynoutrin on up-regulating the expression of S100A1 protein (*p* < 0.05 vs. Reynoutrin group).

Permanent LAD-ligation resulted in a significant up-regulation of MMP2, MMP9, p-p65, and TGF-β1 expression levels in rat myocardial tissue (*p* < 0.05 vs. Sham group). After treatment with reynoutrin, the expression levels of MMP2, MMP9, p-p65, and TGF-β1 in the myocardial tissue of rats with IHF were significantly down-regulated (*p* < 0.05 vs. IHF group). However, the abovementioned effects of reynoutrin on protein expressions were significantly reversed after S100A1 was knocked down (*p* < 0.05 vs. Reynoutrin group). In addition, after knocking down S100A1 alone, the expression levels of MMP2, MMP9, p-p65, and TGF-β1 in the myocardial tissue of IHF rats were significantly up-regulated compared to the IHF group (*p* < 0.05).

**FIGURE 8:**
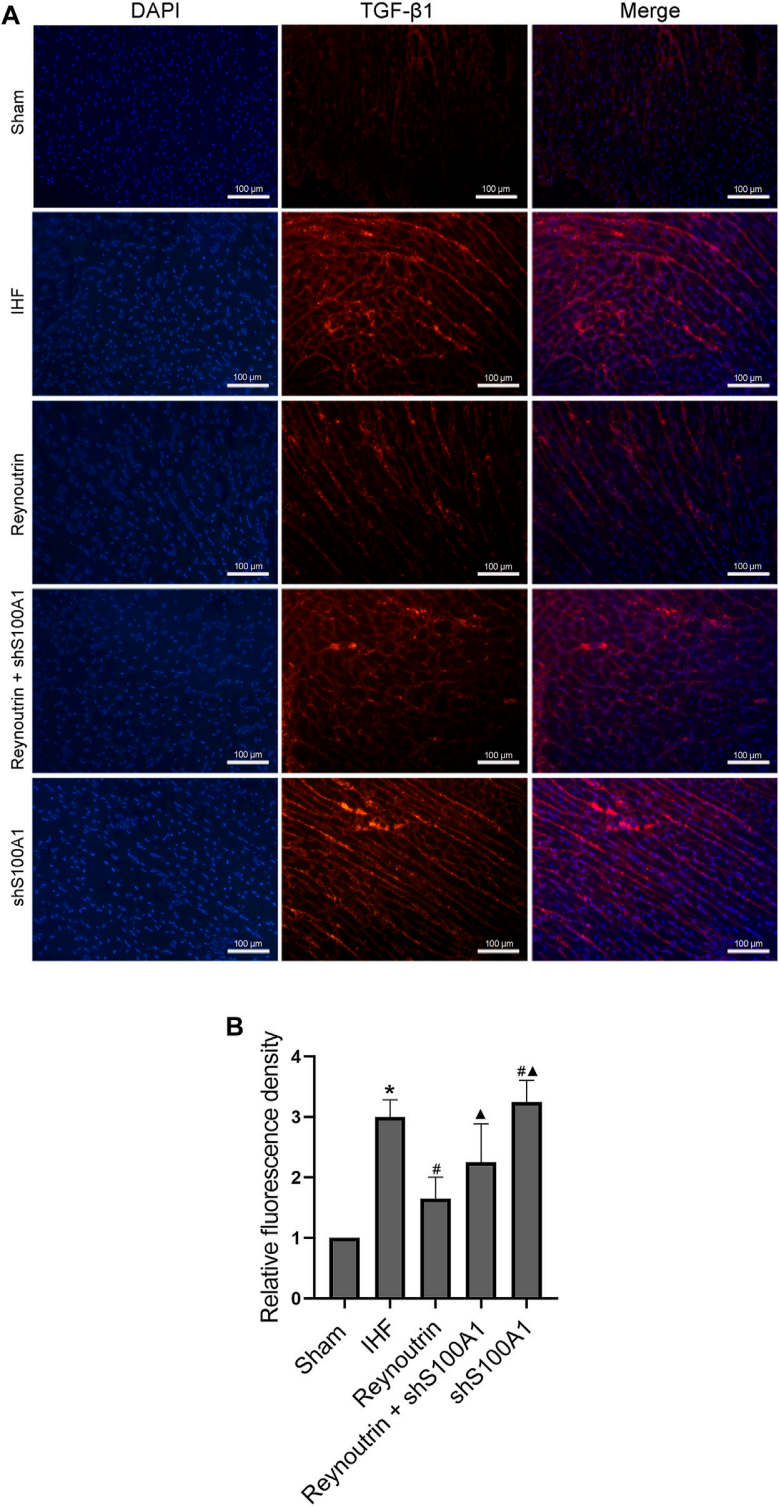
**Effects of reynoutrin and S100A1 knock-down on TGF-β1 expression in paraffin sections of myocardial tissues.** Immunofluorescence staining was used to observe the expression and distribution of TGF-β1 expression in paraffin sections of myocardial tissues. The TGF-β1 emited red under the red excitation light of fluorescence microscope. **(A)** The representative images of immunofluorescence staining of each group. **(B)** The histogram of relative fluorescence density in each experimental group. The values presented as the means ± standard error in each experiment group (*n* = 10). ^*^ *p* < 0.05 vs. Sham group; ^#^ *p* < 0.05 vs. IHF group; ^▲^ *p* < 0.05 vs. Reynoutrin group.

Furthermore, immunofluorescence staining revealed the expression and distribution of TGF-β1, a protein directly involved in myocardial fibrosis, in the left ventricular myocardium of rats in each experimental group. Compared to the sham operation group, higher levels of TGF-β1 protein expression could be detected in the myocardial tissue of rats in the IHF group (*p* < 0.05), while the TGF-β1 protein level in the myocardial tissue of rats with heart failure treated with reynoutrin was significantly reduced (*p* < 0.05 vs. IHF group), as seen in Figure 8. However, after S100A1 was knocked down, the effect of reynoutrin on the TGF-β1 protein level was significantly reversed (*p* < 0.05). Furthermore, knocking down S100A1 alone further induced protein expression of TGF-β1 in myocardial tissue (*p* < 0.05 vs. IHF group).

## Discussion

At present, there are limited studies on reynoutrin. A study by Ho et al. isolated and identified reynoutrin from *Psidium cattleianum* J. Sabine (Myrtaceae), a traditional medicinal plant in French Polynesia, and discovered that it has antioxidant and free radical scavenging properties (Ho et al., 2012). Rehman et al. reported that reynoutrin has certain anti-hepatitis C virus activity (Rehman et al., 2018), while Elhawary et al. investigated the extracts from different parts of *Syzygium cumini* (L.) containing reynoutrin and found that the extracts have certain anti-tumor and anti-diabetic properties; however, the status of reynoutrin in the extracts was not evaluated (Elhawary et al., 2020). Permanent coronary artery ligation leads to myocardial ischemic injury in rats, which is similar to the occurrence of human myocardial infarction caused by rupture of the atherosclerotic plaque (Thygesen et al., 2018). Since the main feature of myocardial infarction in mice is ventricular dilation, their heart failure is similar to human heart failure with reduced ejection fraction (HFrEF) (Hinrichs et al., 2018). In this study, we discovered that reynoutrin improved IHF caused by permanent LAD-ligation in rats. This was achieved by reducing infarct size, inhibiting cardiomyocytes apoptosis, alleviating the degree of myocardial fibrosis, and suppressing the release of inflammatory factors and oxidative stress. In addition, the different doses of reynoutrin used in this study had no obvious toxic effects on cardiomyocytes and normal rats. Our findings indicate that reynoutrin is a potential drug for the prevention and treatment of IHF.

Prolonged ischemia caused by myocardial infarction leads to the death of cardiomyocytes, releasing alarm signals, such as mitochondrial DNA and S100 protein (Frangogiannis, 2018). Then, sentinel cells, such as resident macrophages, endothelial cells, and white blood cells are alerted. The main pattern recognition receptors (PRRs) of cardiac sentinel cells include a variety of Toll-like receptors (TLRs), advanced glycation end product receptors (RAGE), and NOD-like receptors (NLRs) (Hinrichs et al., 2018). The downstream signals of PRRs activate nuclear factor kappa-B (NF-κB), which promotes the expression of chemokines, cytokines, and cell adhesion molecules (CAMs) in the infarct wall (Hinrichs et al., 2018). Proinflammatory cytokines, such as TNF-α, IL-1β, IL-6, and IL-18, amplify the inflammatory response by activating resident and invading effector cells, as well as promote cardiomyocyte apoptosis (Prabhu and Frangogiannis, 2016). Increased expression of MMPs, particularly MMP2 and MMP9, promotes the release of TGF-β1, inducing fibroblasts to differentiate into myofibroblasts by enhancing the expression of SMAD3-dependent *α*-SMA in cardiac fibroblasts, participating in the inflammatory response of injury, depositing a large amount of extracellular matrix protein during the proliferation phase, and finally resulting in pathological fibrosis (Bacmeister et al., 2019; Mouton et al., 2018). Furthermore, ischemic injury can also impair mitochondrial function, leading to excessive oxidative stress, insufficient synthesis of antioxidants such as SOD, and increased production of harmful products such as MDA, which interacts with inflammation and apoptosis, aggravating myocardial injury and fibrosis (Dong et al., 2016; Mukhopadhyay et al., 2018). In this study, we discovered that reynoutrin significantly inhibited the release of TNF-α and IL-6, increased the level of SOD, inhibited the level of MDA, and significantly alleviated apoptosis and fibrosis of the myocardium in rats with IHF. All these indicated that reynoutrin has significant anti-inflammatory, anti-oxidative stress, anti-apoptosis, and anti-fibrosis effects in IHF. Western blotting results showed that LAD-ligation caused a decrease in S100A1 protein expression in the myocardial tissue of the rats, whereas reynoutrin significantly up-regulated the expression of S100A1 and down-regulated the expressions of MMP2, MMP9, p-p65, and TGF-β1. These results suggested that the inhibitory effects of reynoutrin on inflammation, oxidative stress, cardiomyocyte apoptosis, and myocardial fibrosis may be related to its ability to up-regulate the S100A1 protein.

S100 protein participates in the regulation of cellular proliferation, differentiation, apoptosis, and Ca^2+^. Its interaction with various target proteins such as enzymes, cytoskeleton subunits, receptors, transcription factors, and nucleic acids can result in homeostasis, energy metabolism, inflammation, and migration or invasion (Duarte-Costa et al., 2014). Certain S100 proteins are also present in serum and other biological fluids during pathological processes and are used as disease markers (Gonzalez et al., 2020). A growing number of studies have shown that the S100 protein can be used as a potential target for the treatment of various diseases (Gonzalez et al., 2020). S100A1, a member of the S100 protein family, is involved in the regulation of Ca^2+^ homeostasis and is an important regulator of myocardial contraction (Rohde et al., 2010). Among the S100 family, S100A1 has the highest concentration in the myocardium, and its protein concentration level determines the likelihood of heart failure and contractile function following myocardial infarction (Rohde et al., 2014). Studies have shown that an elevated plasma S100A1 level is a significant predictor of ST-segment elevation myocardial infarction, while there is reduced S100A1 expression in failing and hypertrophic heart tissues (Fan et al., 2019). The restoration of S100A1 expression to normal levels in failing cardiomyocytes using gene therapy can improve myocardial contractile function (Bennett et al., 2014; Brinks et al., 2011). In animal experiments, overexpression of S100A1 improved the cardiac function of IHF animal models, whereas a lack of S100A1 led to increased arterial pressure and mortality after myocardial infarction in mice (Jungi et al., 2018; Pleger et al., 2011; Soltani et al., 2021). However, the specific mechanism of S100A1 involved in the pathophysiological regulation of IHF is still not fully understood. In this study, we found that knocking down the expression of S100A1 significantly attenuated the effects of reynoutrin on improving cardiac function, cardiomyocytes apoptosis, myocardial inflammation and fibrosis, and down-regulating the expressions of MMP2, MMP9, p-p65, and TGF-β1 in rats with IHF. The findings indicated that reynoutrin inhibited MMP2 and MMP9 expressions, as well as NF-κB transcription activity by targeting S100A1, thereby alleviating myocardial inflammation and oxidative stress, reducing infarct size and cardiomyocytes apoptosis, reducing myocardial fibrosis, and show improvements in heart failure caused by ischemia.

In conclusion, reynoutrin is a potential natural drug for the treatment and improvement of IHF, which showed a significant therapeutic effect in rats with heart failure caused by permanent LAD-ligation. The effect of reynoutrin on improving IHF is related to its ability in improving myocardial inflammation, inhibit oxidative stress, reduce infarct size and cardiomyocytes apoptosis, and improve myocardial fibrosis. This study further confirmed that S100A1 is a potential target of reynoutrin, and that knocking down the expression of S100A1 in the myocardium can significantly reverse the therapeutic effect of reynoutrin. We believe that the anti-IHF effect of reynoutrin can be achieved by up-regulating the expression of S100A1, which inhibits the expressions of MMP2 and MMP9, as well as the transcriptional activity of NF-κB. However, this study lacks of positive control and is not comprehensive enough to explore the process of reynoutrin improving inflammation and remodeling after myocardial infarction. In addition, the molecular mechanism of reynoutrin is also not in-depth enough. We should further explore the protective effects of reynoutrin on cardiomyocytes *in vitro*, combined with *in vivo* research, to provide more comprehensive data support for the positive effects of reynoutrin in improving IHF.

## Data Availability

The raw data supporting the conclusions of this article will be made available by the authors, without undue reservation.
