# Supplementary figures and images for: Reynoutrin Improves Ischemic Heart Failure in Rats Via Targeting S100A1
Source: Front Pharmacol. 2021 Jul 23;12:703962. doi: 10.3389/fphar.2021.703962 (PMC8343003; doi:10.3389/fphar.2021.703962)

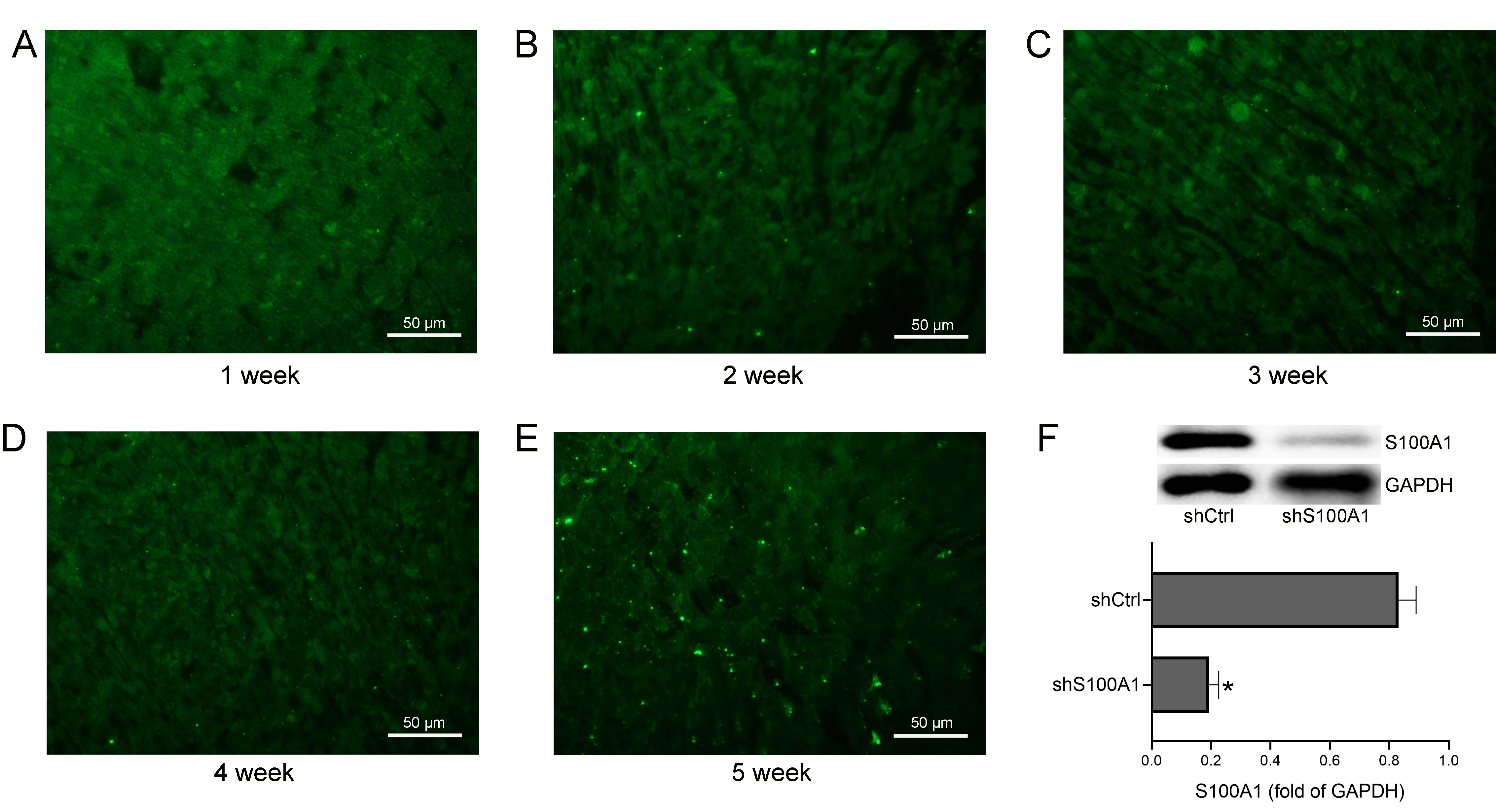

Supplement: Supplementary file 1 [file Image2.TIF]

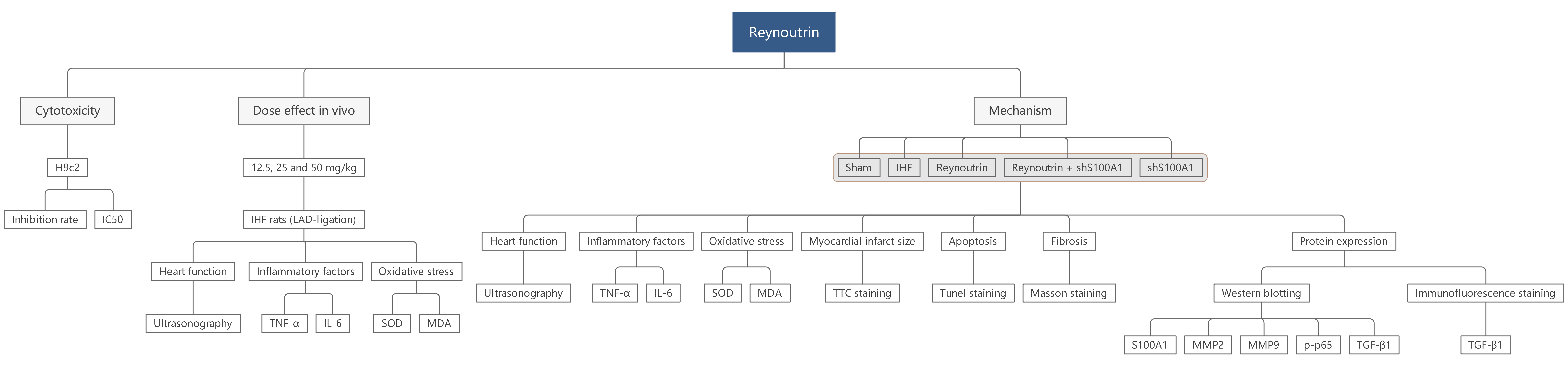

Supplement: Supplementary file 2 [file Image1.TIF]
